# Osteoporosis and its associated factors among patients attending Manakamana Hospital, Chitwan, Nepal

**DOI:** 10.1371/journal.pone.0289517

**Published:** 2024-02-21

**Authors:** Shankar Dhakal, Kalpana Sharma, Kishor Adhikari, Alisha Joshi, Sunita Poudyal

**Affiliations:** 1 Manakamana Hospital, Bharatpur, Chitwan, Nepal; 2 Schools of Nursing, Chitwan Medical College, Bharatpur, Chitwan, Nepal; 3 School of Public Health & Dept. of Community Medicine, Chitwan Medical College, Chitwan, Nepal; Pokhara University, NEPAL

## Abstract

**Background:**

Osteoporosis is the most common skeletal disorder that weakens bones and increases their susceptibility to fractures. It is becoming an urgent and serious global epidemic. Early diagnosis and treatment are essential to reduce morbidity and mortality associated with it. This study aimed to find out the prevalence of osteoporosis among patients attending at Manakamana Hospital, Bharatpur, Chitwan, Nepal.

**Methods:**

A cross-sectional study was adopted and 623 patients attending at orthopaedic outpatients department (OPD) of Manakamana Hospital were selected using non-probability consecutive sampling technique. Data were collected from 15^th^ October 2021 to 15^th^ April, 2022, by using interview schedule, chart review and Bone Mineral Density (BMD) measurement through calcaneal ultrasonography. Ethical approval was obtained from Nepal Health Research Council Ethical Review Board prior to study procedures. Obtained data were analysed using descriptive statistics. Association between the variables were measured using chi-square test.

**Results:**

The mean age of the patients was 43.5 (±14.26) years. Nearly half (44%, n = 274) were middle aged adults, 59.7% were female and 56.0% were involved in agriculture and household chores. Nearly half of the patients (45.7%) were overweight/ obese, 7.9% were smokers and 13.5% had habit of alcohol use. Osteopenia or low bone density was detected in 58.9% patients and 19.4% had osteoporosis. The prevalence of osteoporosis was significantly associated with age group (p = <0.001) and educational status (p = 0.013) of the patients.

**Conclusions and recommendations:**

Osteoporosis and osteopenia are prevalent in patients attending in the hospital. Hence, awareness, early screening, and treatment are necessary for the hospital attended patients to enhance their health and, minimize the risk of osteoporosis and the consequences associated with it.

## Introduction

Osteoporosis is the most common skeletal disorders that weakens bones and increases their susceptibility to fractures. It is becoming an urgent and serious global epidemic which accounts 18.3% of the world population [1]. Asia has higher prevalence of osteoporosis (24.3%) than the USA, and Australia but lower prevalence than Africa and Europe [1]. Currently it is estimated that approximately 500 million people worldwide suffer from this disease [2, 3]. Osteoporotic fractures are characterized as fractures associated with low Bone Mineral Density (BMD) [4]. Quantitative Ultrasonography (QUS) is a quick and reliable method for identifying osteoporosis in both men and women in developing nations [5].

In Nepalese population, the prevalence of osteoporosis is high [6–8] and studies showed variation on prevalence from 8.2% [7] to 22.4% [6]. Many factors such as sex, age, smoking habit [9, 10], lower Body Mass Index (BMI), lower daily calcium consumption [9], wrist fracture, and spinal deformities [10] are significantly associated with the osteoporosis.

Unfortunately, osteoporosis is often undiagnosed until a fracture occurs. Osteoporotic fractures have significant, long-lasting impacts on mortality, increase in functional limitations and decreases in quality of life, and place a significant financial and human resource burden on healthcare systems [11]. One systematic review showed that the expense of osteoporosis places a heavy financial strain on nation’s healthcare system throughout the world. Fracture-related expenses were the primary cost drivers while, indirect costs included lost productivity and incapacity. The health system’s expenses can be greatly decreased by preventing osteoporosis and it’s consequences [12]. Few studies have been conducted in Nepalese population [6–9] on prevalence of osteoporosis and these studies were conducted in different hospitals of Nepal [6], Kathmandu [7, 9] and Chitwan [8] and they were focused on middle aged women [8] and 50 years and older population [9] as well as variation in findings related to prevalence of osteoporosis among 20 years and above aged groups [6, 7]. Hence, this study aimed to assess the prevalence of osteoporosis among patients attending Manakamana Hospital, Chitwan, Nepal.

## Material and methods

### Study design, period and setting

A cross-sectional study was conducted among the patients attending Orthopaedic Out Patients Departments of Manakamana Hospital, Chitwan, Nepal from August 2021 to July 2022. This Manakamana hospital is 100 bedded private hospital located in central Nepal where highly qualified orthopaedic consultants are available and patients from different parts of the country visit for the treatment. In addition, this hospital has health insurance facility. Data were collected from 15^th^ October 2021 to 15^th^ April, 2022.

### Study population and eligibility criteria

Those patients who were visited to the Out Patient Department (OPD) of Manakamana hospital for the orthopaedic consultation during data collection period were the study population. This study included patients aged 20 years and above, visiting the hospital during data collection period and willing to participate in the study whereas those patients who had previous history of fracture, pregnant and severely ill or unable to communicate were excluded from the study.

### Sample size determination and sampling technique

Sample size was calculated for the study using Cochrane’s formula (n = z^2^pq/d^2^) considering 95% confidence interval (z = 1.96) and 4% allowable error (d = 0.04). Assuming a p of 0.373 (37.3% prevalence of osteoporosis in Nepal) [9], the minimum required calculated sample size was 562. With addition of 10% non-response rate (57), final sample size was 619 = 623.

Non-probability consecutive sampling technique was used to select the desired study sample because it is the best method of non-probability sampling for controlling sampling bias. Patients who met the inclusion criteria and attended in the OPD during study periods were taken as study sample and they were taken till desired number achieved.

### Data collection tools and measurement

Data were collected by the researchers using structured interviews schedule, bio-physiological measurement and records reviews on the day of hospital visit. First, patients were identified from their Out Patients Department (OPD) ticket which was issued from the OPD counter of the hospital upon patients request for orthopaedic service. Researchers contacted the patients in the orthopaedic OPD. The purpose of the study was explained to them. Data related to socio-demographic (age, sex, education, occupation) and personal habit (smoking, alcohol habit) were collected using a structured interview schedule. Patient was instructed to wear light clothes and remove shoes. Then weight and height were measured through weighing scale and stadimetry. Then patient was kept comfortably in sitting position in chair besides the SONAST machine. Using a mercury sphygmomanometer (model BK1005), blood pressure was taken twice 10 minute apart in accordance with 2020 International Society of Hypertension Guidelines for Global Hypertension Practice and mean reading was calculated. After that, bone mineral density (BMD) was measured at calcaneus (heel bone) using portable Ultrasound bone Densimeter (SONOST 2000) machine. Patient was asked to place his/her bare foot in the machine firmly. Once machine was started, the numerical value of the BMD had shown in the screen and it was recorded. After completion, result was briefly explained to the patients by the consultant and needed treatment was given. The measurement of BMD was done on the right heel for all patients. The examinations were performed by a single trained professional. The apparatus was calibrated daily.

### Data quality control

Nepali version questionnaire were used for the data collection. Measurements were taken using standard protocol. Each anthropometric parameter was taken twice and the measurement was repeated if the difference existed. One hour briefing session was arranged on the data collection process for the data collectors and technician. Trained technician was used to measure BMD.

### Data management and analysis

The collected data were coded and entered into IBM SPSS^®^ software version 20 for window. Continuous variables were presented as mean, standard deviation, median, and interquartile range while categorical variables were presented as the frequency and percentage. The prevalence of osteoporosis was estimated through WHO criteria for osteoporosis (normal BMD if T-score >-1 SD, osteopenia if T-score is -1 SD to -2.5 SD, and osteoporosis if T-score <-2.5 SD). The T-score represents numbers that compare the condition of bone with those of an average young adult with healthy bones. It is easy to use and provide an excellent estimation of the risk of osteoporotic fracture. BMI was calculated as weight in kilograms/ height in meter square. Chi-square test was applied to measure the association between prevalence of osteoporosis and selected variables. All the statistical significance was set at p<0.05.

## Results

Out of 623 respondents, highest proportion (44.0%) of respondents was middle aged adults. Majorities of the respondents were female (59.7%), completed secondary and above level of education (89.4%), and involved in agriculture and household chores (56.0%). Few respondents were smokers (7.9%) and had habit of alcohol use (13.5%). Nearly half (45.8%) of respondents were overweight and obese and few had high systolic BP (6.4%) and diastolic BP (11.2%) (Table 1).

**Table 1:** Socio-demographic and personal habit of respondents n = 623.

Variable	Number	Percent
**Age (completed in years)**		
Young (20–39 year)	262	42.1
Middle (40–59 year)	274	44.0
Elderly (≥ 60 year)	87	14.0
*Mean (SD) = 43*.*5(±14*.*26)*, *Min-20 year*, *Max-99 year*		
**Sex**		
Male	251	40.3
Female	375	59.7
**Education**		
Illiterate	37	5.9
Basic	29	4.7
Secondary	273	43.8
Bachelor and above	284	45.6
**Occupation**		
Agriculture and Household work	349	56.0
Service	170	27.3
Business	78	12.5
Others (Retired, Students)	26	4.2
**Smoking habit**		
Yes	49	7.9
No	574	92.1
**Alcohol habit**		
Yes	84	13.5
No	539	86.5
**BMI status**		
Underweight (<18.5)	42	6.7
Normal (18.5–24.9)	296	47.5
Overweight (25.0–29.9)	185	29.7
Obesity (30 and above)	100	16.1
**Systolic BP**		
Normal (<120 mm of Hg)	277	44.5
Pre-hypertensive (120–139 mm of Hg)	306	49.1
Hypertensive (>140 mm of Hg)	40	6.4
**Diastolic BP**		
Normal (<80 mm of Hg)	307	49.3
Pre-hypertensive (80–89 mm of Hg)	246	39.5
Hypertensive (>90 mm of Hg)	70	11.2

Out of 623 respondents, more than half (59.9%) respondents had osteopenia or low bone density and nearly one fifth (19.4%) had osteoporosis (Table 2).

**Table 2:** Prevalence of osteoporosis among the respondents n = 623.

Osteoporosis status	Number	Percent
Normal bone density (T-score is equal to or above -1 S.D).	135	21.7
Osteopenia or low bone density (T-score ranges between -1.1 S.D. and -2.5 S.D),	367	58.9
Osteoporosis (T-score is equal to or below -2.5 S.D).	121	19.4

Prevalence of osteoporosis was significantly associated with age (*p*<0.001) and education (*p*<0.013) of the respondents whereas marginally significant association was found with smoking habit. It indicates that the osteoporosis is higher among elderly and illiterate respondents compared to other age group and educated people. However other variables were not associated with it (Table 3).

**Table 3:** Association between prevalence of osteoporosis and selected variables n = 623.

Variables	Prevalence osteoporosis			x^2^	*p- value*
	Normal No. (%)	Osteopenia No. (%)	Osteoporosis No. (%)		
**Age group**					
Young	62 (23.7)	170 (64.9)	30 (11.5)	70.963	**<0.001**
Middle	65 (23.7)	163 (59.5)	46 (16.8)		
Elderly	8 (9.2)	34 (39.1)	45 (51.7)		
**Sex**					
Male	63 (25.1)	140 (55.8)	48 (19.4)	3.002	0.223
Female	72 (19.4)	227 (61.0)	73 (19.6)		
**Education**					
Illiterate	9 (24.3)	14 (37.8)	14 (37.8)	16.140	**0.013**
Basic	5 (17.2)	20 (69.0)	4 (13.8)		
Secondary	71 (26.0)	157 (57.5)	45 (16.5)		
Bachelor and above	50 (17.6)	176 (62.0)	58 (20.4)		
**Occupation**					
Business	21 (26.9)	46 (59.0)	11 (14.1)	4.102	0.663
Farmer/agriculture	70 (20.1)	204 (58.5)	75 (21.5)		
Service	39 (22.9)	100 (58.8)	31 (18.2)		
Other (retired/student)	5 (19.2)	17 (65.4)	4 (15.4)		
**Smoking habit**					
No	119 (20.7)	346 (60.3)	109 (19.0)	5.798	**0.050**
Yes	16 (32.7)	21 (42.9)	12 (24.5)		
**Alcohol habit**					
No	111 (20.6)	323 (59.9)	105 (19.5)	2.702	0.259
Yes	24 (28.6)	44 (52.4)	16 (19.0)		
**BMI status**					
Underweight	11 (26.2)	17 (40.5)	14 (33.3)	7.163	0.128
Normal	63 (21.3)	180 (60.8)	53 (17.9)		
Overweight/obese	61 (21.4)	170 (59.6)	54 (18.9)		

## Discussion

This study was aimed to assess the prevalence of osteoporosis and its associated factors among patients attending in hospital. Our findings revealed that nearly one fifth of the patients have osteoporosis. Further, it found age and education as the influencing variables for the osteoporosis.

In this study, prevalence of osteoporosis and osteopenia were 19.4% and 59.9% respectively according to BMD scoring. It is consistent with studies from central part of Nepal [6], global data of osteoporosis [1, 13] and Hongkong [14]. Bagudai and Upadhaya showed 22.4% and 60.6% prevalence of osteoporosis and osteopenia respectively among Nepalese population attending selected hospitals of Pokhara, Chitwan and Bhairahawa [6]. Likewise, systematic review and meta-analysis showed 19.7% and 40.4% global prevalence of osteoporosis and osteopenia respectively [13], whereas it was lower than the studies from Kathmandu, Nepal [9], Panjab, India [15] and United States [10]. Chaudhary and colleagues showed 37.3%, and 38.5% prevalence of osteoporosis and osteopenia respectively among 50 years and above people of Nepal [9]. Likewise, study in India revealed the prevalence of osteoporosis and osteopenia among 30.5% and 44.2% respectively, in postmenopausal women of Punjab [15]. The discrepancy rates of osteoporosis in the studies might be due to different nature of population included in the studies. In our study we included 20 years of aged population whereas other studies included 50 years and older population [9, 10] and postmenopausal women [15].

Osteoporosis varies among population to population according to their age [16]. In our study, osteoporosis is higher among middle aged and elderly population compared to young adult. It is similar with other studies conducted in Nepal [6, 7, 9, 10, 17] and China [18]. This might be due to the facts that aging is associated with decreases in the growth hormone secretion from the anterior pituitary and decreased systemic and local skeletal production of IGF-1 and IGF-2, growth factor binding proteins which may contribute to age-related bone loss [19]. Middle aged groups are main workforce. If they suffered from osteoporotic related fractures, they will not be able to report to their job, and moreover, they need to spend money for treatment. Likewise, elderly population that suffer from osteoporotic related fractures and its complications, that too will put a lot of strain on healthcare spending aside from morbidity suffered by the patients. Hence, proper health care planning and targeted interventions are of utmost importance to minimize the menace of osteoporosis.

The present study found that the prevalence of osteoporosis is higher among illiterate participants or with lower education levels compared to people with higher education level and education status is significantly associated with the prevalence of osteoporosis. This finding agreed with the studies conducted in Northwestern China [17] and morocco [20] which reported the significant association between educational level and risk of osteoporosis. This finding is in line with the western countries finding in which less educated women are more prone to low density bone and osteoporosis than highly educated women [21–23]. The reasons behind might be due to the facts that highly educated people may have more knowledge on osteoporosis and hence involved in the greater level of physical activity and proper nutritional intake compared to low educated participants.

Inspite of publics misconception on osteoporosis with women, it can affect both men and women equally [24] and our finding is consistent with it showing that there was no significance difference on the prevalence of osteoporosis between women and men.This is also supported by the hospital based study conducted in central part of Nepal among hospital attended healthy patients aged 20 years and above [25]. However, other studies from Italy [25] and China [18, 26] have shown the higher incidence of osteoporosis in women compared to men. It is due to the fact that women had lower vitamin D, and higher bone remodeling markers compared to men [25]. Likewise, a systematic review and meta-analysis [1] revealed higher prevalence of osteoporosis among women in the world compared to men (women-23.2%, men-11.7%). Further, evidence showed that the bone thinning begins in both men and women between the ages of 35 and 40. In contrast to women, who experience extra bone loss associated with oestrogen insufficiency during peri-menopausal and post-menopausal periods, men are seen to experience a minor longitudinal bone loss throughout life [27].

In our study, smoking habit was marginally significant with the osteoporosis and this finding is almost consistent with the findings of the studies done in USA [10], India [15] and China [17] which found smoking as independent predictor of low bone mineral density. We found that there was no significant association between the prevalence of osteoporosis with the occupation, and alcohol consumption of the patients. In contrast to this, findings from Nepal [9], USA [10] and China [17] have shown that alcohol consumption, physical activity [17] and daily dietary calcium intake [9] as other modifiable risk factors of osteoporosis. This might be due to difference in sample characteristics (ie. age group) in the studies, because patients included in our study were 20 years and above age group whereas other studies included people with higher age groups.

Several studies had supported that the higher body mass index (BMI) is a protective factor for osteoporosis [9, 15, 20] BMI and BMD are positively associated [8, 28]. Patients with high BMI had a higher BMD and a lower risk of osteoporosis than those with a normal BMI [29]. Our study revealed that BMI is not statistically associated with the prevalence of osteoporosis. Unlike this finding, other studies [6, 10, 17] revealed the significant negative relationship between BMI and osteoporosis. The difference in findings might be due to difference in physical activity, sun exposure and dietary intake habit of study subjects included in the studies.

This study has some limitations and strength to note. Owing to cross-sectional study design, this was unable to establish cause and effects relationship. Additionally, patients were selected from orthopaedic OPD of a hospital using non-probability sampling; general prevalence could be different. Furthermore, inability to explore the data on other risk factors like co-morbidities, drugs, and nutritional status, vitamin D, calcium, thyroid and related hormones, oestrogen and its various receptors could be another limitation of this study. However, our findings are reflective of prevalence of osteoporosis among patients attending to orthopaedic OPD because of relatively large sample size. Also using portable DEXA scan aligns our research with current international osteoporosis foundations guidelines for evaluating osteoporosis. These estimates could be helpful in determining priorities, formulating policies, and allocating resources for osteoporosis prevention and treatment through education, screening and collaborative care. Large scale, multi-centric, randomized sampling study is needed to minimize the bias and establish the real burden of the problem.

## Conclusion

Nearly one fifth of the patients visiting orthopaedic OPD have osteoporosis while more than half have osteopenia. Patients’ age and education status tend to influence the prevalence of osteoporosis. Hence, this study suggests that every patients attending in a hospital should be tested for osteoporosis to minimize the possible fracture risk and its consequences, so that better health care can be assured. Furthermore, healthcare workers and policymakers need to organize the awareness raising programs on osteoporosis for the risk groups (especially middle aged and elderly individuals with lower education levels) to enhance their knowledge regarding osteoporosis and its preventive measures.

## Supporting information

S1 File The questionnaire used for the data collection. (DOCX)

## References

[1] Salari N, Ghasemi H, Mohammadi L, Behzadi MH, Rabieenia E, Shohaimi S, et al. The global prevalence of osteoporosis in the world: a comprehensive systematic review and meta-analysis. J Orthop Surg Res. 2021 Oct 17;16(1):609. doi: 10.1186/s13018-021-02772-0 ; PMCID: PMC8522202. 34657598 PMC8522202

[2] International Osteoporosis Foundation. Epidemiology of osteoporosis and fragility fracture. 2023. https://www.osteoporosis.foundation/facts-statistics/epidemiology-of-osteoporosis-and-fragility-fractures

[3] International Osteoporosis Foundation. Key Statistics for Asia. https://www.osteoporosis.foundation/facts-statistics/key-statistic-for-asia

[4] Osteoporosis: assessing the risk of fragility fracture. London: National Institute for Health and Care Excellence (NICE); 2017 Feb. (NICE Clinical Guidelines, No. 146.).https://www.ncbi.nhm.nih.gov/books/NBK554920/32186835

[5] Kung AW, Ho AY, Ross PD, Reginster JY. Development of a clinical assessment tool in identifying Asian men with low bone mineral density and comparison of its usefulness to quantitative bone ultrasound. Osteoporos Int. 2005 Jul;16(7):849–55. doi: 10.1007/s00198-004-1778-z . 15611839

[6] Bagudai S, & Upadhayay HP. Prevalence of osteoporosis and osteopenia status among Nepalese population using calcaneal ultrasonography method. JCMS Nepal. 2019;15(4): 249–255. 10.3126/jcmsn.v15i4.24008

[7] Shrestha S, Dahal S, Bhandari P, Bajracharya S, & Marasini A. Prevalence of osteoporosis among adults in a tertiary care hospital: a descriptive cross-sectional study. J Nepal Med Assoc. 2019 Nov-Dec 31;57(220): 393. doi: 10.31729/jnma.4753 ; PMCID: PMC7580411. 32335647 PMC7580411

[8] Dhakal KS, Dhakal S, Aryal B. Prevalence of osteoporosis among middle aged women in Chitwan District of Nepal. IJPBA. 2012; 3(4):779–82.

[9] Chaudhary NK, Timilsena MN, Sunuwar DR, Pradhan PMS, & Sangroula RK. Association of lifestyle and food consumption with bone mineral density among people aged 50 years and above attending the hospitals of Kathmandu, Nepal. J Osteoporosis. 2019 May 22; 2019:1536394. doi: 10.1155/2019/1536394 ; PMCID: PMC6556264. 31240093 PMC6556264

[10] Rozental TD, Shah J, Chacko AT, & Zurakowski D. Prevalence and predictors of osteoporosis risk in orthopaedic patients. Clin Orthop Relat Res. 2010 Jul; 468(7):1765–72. doi: 10.1007/s11999-009-1162-6 ; PMCID: PMC2881983. 19911243 PMC2881983

[11] Cauley JA. Public health impact of osteoporosis. J Gerontol A Biol Sci Med Sci. 2013 Oct; 68(10): doi: 10.1093/gerona/glt093 ; PMCID: PMC3779634. 23902935 PMC3779634

[12] Rashki Kemmak A, Rezapour A, Jahangiri R, Nikjoo S, Farabi H, Soleimanpour S. Economic burden of osteoporosis in the world: A systematic review. Med J Islam Repub Iran. 2020 Nov 12;34:154. doi: 10.34171/mjiri.34.154 ; PMCID: PMC7787041. 33437750 PMC7787041

[13] Xiao PL, Cui AY, Hsu CJ, Peng R, Jiang N, Xu XH, et al. Global, regional prevalence and risk factors of osteoporosis according to the World Health Organization diagnostic criteria: a systematic review and meta-analysis. Osteoporos Int. 2022 Oct; 33(10): 2137–2153. doi: 10.1007/s00198-022-06454-3 . 35687123

[14] Lo SS. Prevalence of osteoporosis in elderly women in Hong Kong. Osteoporos Sarcopenia. 2021 Sep;7(3):92–97. doi: 10.1016/j.afos.2021.09.001 Epub 2021 Sep 9. ; PMCID: PMC8486614. 34632111 PMC8486614

[15] Khinda R, Valecha S, Kumar N, Walia JPS, Singh K, Sethi S, et al. Prevalence and predictors of osteoporosis and osteopenia in postmenopausal women of Punjab, India. Int J Environ Res Public Health. 2022 Mar 4;19(5):2999. doi: 10.3390/ijerph19052999 ; PMCID: PMC8910053. 35270692 PMC8910053

[16] Lee J, Lee S, Jang S, Ryu OH. Age-related changes in the prevalence of osteoporosis according to gender and skeletal site: The Korea National Health and Nutrition Examination Survey 2008–2010. Endocrinol Metab (Seoul). 2013 Sep;28(3):180–91. doi: 10.3803/EnM.2013.28.3.180 ; PMCID: PMC3811701. 24396677 PMC3811701

[17] Tian L, Yang R, Wei L, Liu J, Yang Y, Shao F, et al. Prevalence of osteoporosis and related lifestyle and metabolic factors of postmenopausal women and elderly men: A cross-sectional study in Gansu province, Northwestern of China. Medicine. 2017; 96(43). doi: 10.1097/MD.0000000000008294 ; PMCID: PMC5671832. 29068999 PMC5671832

[18] Mengmeng Z, Yagang L, Ying L, Xuena P, Binbin L, & Liu Z. A study of bone mineral density and prevalence of osteoporosis in Chinese people of Han nationality from Changchun. Arch Osteoporos. 2012; 7: 31–36. doi: 10.1007/s11657-011-0066-8 . 23225279

[19] Demontiero O, Vidal C, Duque G. Aging and bone loss: new insights for the clinician. Ther Adv Musculoskelet Dis. 2012 Apr;4(2):61–76. doi: 10.1177/1759720X11430858 ; PMCID: PMC3383520. 22870496 PMC3383520

[20] Allali F, Rostom S, Bennani L, Abouqal R, Hajjaj-Hassouni N. Educational level and osteoporosis risk in postmenopausal Moroccan women: a classification tree analysis. Clin Rheumatol. 2010 Nov; 29(11):1269–75. doi: 10.1007/s10067-010-1535-y Epub 2010 Jul 30. . 20676712

[21] Deter S, Leslie WD, Reed M, Lix L, & MacWilliam L. The effect of socio-economic status on bone density testing in a public health-care system. Osteoporos Int. 2007 Feb; 18 (2): 153–8. doi: 10.1007/s00198-006-0212-0 . 17019518

[22] Brennan SL, Pasco JA, Urquhart DM, Oldenburg B, Wang Y, & Wluka A E. Association between socio-economic status and bone mineral density in adults: a systematic review. Osteoporos Int. 2011 Feb; 22(2): 517–27. doi: 10.1007/s00198-010-1261-y . 20449573

[23] Maddah M, Sharami SH, & Karandish M. Educational difference in the prevalence of osteoporosis in postmenopausal women: a study in northern Iran. BMC Public Health. 2011 Nov 3; 11(845):1–3. doi: 10.1186/1471-2458-11-845 ; PMCID: PMC3229620. 22054508 PMC3229620

[24] Sözen T, Özışık L. Başaran Nursel Çalık. An overview and management of osteoporosis. Eur J Rheumatol. 2017 Mar; 4(1): 46–56. doi: 10.5152/eurjrheum.2016.048 PMCID: PMC5335887. 28293453 PMC5335887

[25] De Martinis M, Sirufo MM, Polsinelli M, Placidi G, Di Silvestre D, Ginaldi L. Gender differences in osteoporosis: A single-center observational study. World J Mens Health. 2021 Oct; 39(4):750–759. doi: 10.5534/wjmh.200099 Epub 2020 Nov 26. ; PMCID: PMC8443988. 33474849 PMC8443988

[26] Wang L., Yu W., Yin X., Cui L., Tang S., Jiang N., et al. Prevalence of osteoporosis and fracture in China: the China osteoporosis prevalence study. JAMA Netw Open. 2021 Aug 2;4(8): e2121106. doi: 10.1001/jamanetworkopen.2021.21106 ; PMCID: PMC8369359. 34398202 PMC8369359

[27] Kadam NS, Chiplonkar SA, Khadilkar AV, & Khadilkar VV. Prevalence of osteoporosis in apparently healthy adults above 40 years of age in Pune City, India. Indian J Endocrinol Metab. 2018 Jan-Feb; 22(1): 67–73. doi: 10.4103/ijem.IJEM_438_17 ; PMCID: PMC5838914. 29535940 PMC5838914

[28] Ma M, Feng Z, Liu X, Jia G, Geng B, & Xia Y. (2021). The saturation effect of body mass index on bone mineral density for people over 50 years old: a cross-sectional study of the US population. Front Nutr. 2021 Oct 15; 8: 763677. doi: 10.3389/fnut.2021.763677 ; PMCID: PMC8554069. 34722617 PMC8554069

[29] Barrera G, Bunout D, Gattás V, de la Maza MP, Leiva L, & Hirsch S. A high body mass index protects against femoral neck osteoporosis in healthy elderly subjects. Nutrition. 2004 Sep; 20(9): 769–71. doi: 10.1016/j.nut.2004.05.014 . 15325685

