# Supplementary material for: Osteoporosis and its associated factors among patients attending Manakamana Hospital, Chitwan, Nepal
Source: PLoS One. 2024 Feb 21;19(2):e0289517. doi: 10.1371/journal.pone.0289517 (PMC10881011; doi:10.1371/journal.pone.0289517)
Supplement: S1 File — (DOCX) [file pone.0289517.s001.docx]

**Patients’ Inclusion Criteria**

| **S.N** | **Criteria** | **Response** | |
| --- | --- | --- | --- |
|  |  | Yes | No |
| 1 | History of Fracture |  |  |
| 2 | Intake of calcium supplement |  |  |
| 3 | Intake of oral contraceptive pill |  |  |
| 4 | Pregnancy test positive |  |  |
| 5 | Severely ill |  |  |
| 6 | Unable to communicate |  |  |

Those who did not have above mentioned conditions were considered as healthy adult and included in the study

**Performa on Bone Mineral Density Measurement of People Aged 20 Years and Above**

Age in Year: ____________________

Sex: Male/Female

Education: Illiterate/No formal education/Basic/Secondary/higher Secondary

Type of family: Nuclear/Joint

Occupation: household work/agriculture/service/business/labour/other specify……………………………………

Marital Status:_______________________________________________

Smoking status: Current/Ex-smoker/Non-smoker

Alcohol consumption: Never/once a week or less/2-6 times per week/everyday

Height (in cm)_______________________________________________

Weight (in kg)_______________________________________________

BMI: Kg/M^2^

BP (mm of Hg): Systolic BP 1st ________ 2^nd^ ________

Diastolic BP 1^st^ ________ 2^nd^ ________

BMD findings: calcaneal bone of right heel (T score):

**Thank You Very Much for Your Kind Cooperation**
